# The translational sciences clinic: From bench to bedside

**DOI:** 10.1017/cts.2020.529

**Published:** 2020-08-25

**Authors:** Perry Halushka, Tammy L. Loucks, Rechelle Paranal, Jillian Harvey, Kristen Briggman, Diana Lee-Chavarria, Carol Feghali-Bostwick

**Affiliations:** 1 Departments of Pharmacology and Medicine, College of Graduate Studies, Medical University of South Carolina, Charleston, SC, USA; 2 South Carolina Clinical and Translational Research Institute, Medical University of South Carolina, Charleston, SC, USA; 3 Department of Healthcare Leadership and Management, College of Health Professions, Medical University of South Carolina, Charleston, SC, USA; 4 Department of Medicine, College of Medicine, Medical University of South Carolina, Charleston, SC, USA

**Keywords:** Clinical exposures, bench to bedside, predoctoral trainees, team-based approach, mentored clinical experience

## Abstract

The mission of the National Center for Advancing Translational Science (NCATS) is to speed the development of drugs from discovery to approval to dissemination and implementation. The Medical University of South Carolina and the South Carolina Clinical and Translational Research Institute host a NCATS funded predoctoral T32 training grant (TL1) with a focus on translational research. Doctoral (PhD) trainees working at the bench usually have limited opportunity for clinical interactions to gain a clinical perspective on the diseases that are the focus of their dissertation research. To provide TL1 trainees with an opportunity to see how their research could be translated into improved patient care, we developed a mentored clinical exposure experience named the Translational Sciences Clinic. Trainees spend one-half day a week in a clinic related to their basic science research for one semester interacting with patients and clinical mentors and discuss the most recent literature related to the patient’s clinical problem with their clinical mentor. Trainees deemed the rotation to be one of the most rewarding experiences that they had as a part of their predoctoral training. Participating clinical mentors were also very enthusiastic and agreed that they would be willing to mentor similar trainees again.

## Introduction

The fundamental underpinning of impactful clinical/translational research is solid basic science research [1]. PhD trainees, while often conducting basic science research that may some day lead to a new therapeutic or diagnostic approach, rarely have the opportunity to see the potential clinical impact that their discovery may have on patients. As a result, there is often not a clear understanding of how the bench research may impact individuals with the condition being studied. Thus, we felt that it was important to create an opportunity for PhD trainees to engage with patients affected by the diseases that they were researching. Through these patient interactions, our goal was to close the divide between basic science and patient care by providing trainees with real-life experiences that may impact how they think about the significance of their research in the future. To provide that experience, we had them participate in the Translational Sciences Clinic. The concept of the Translational Sciences Clinic originated in the Medical University of South Carolina Medical Scientist Training Program (MSTP) approximately 12 years ago, and the offering has been a highly rated translational research experience by both faculty and the MD/PhD students. Thus, we decided to implement this very successful translational/team science activity for the predoctoral TL1 trainees based on the reasoning that this approach would enable predoctoral trainees to experience first-hand how their laboratory-based research could address a clinical or therapeutic need and directly impact patients’ lives. It also highlights the significance of the translational research process and strengthens the trainees’ mentoring teams by incorporating clinical mentors. The experience was structured so that it would not significantly prolong the time for the trainees to obtain their PhD degree.

The TL1 trainees shadow a clinical mentor for one-half day a week in an outpatient clinic. The clinical mentor is a physician who is chosen based on his/her area of practice and demonstrated commitment to mentoring. They are also paired whenever possible with a K scholar to provide a near-peer type mentoring experience. The semester-long course is two credit hours and the clinic in which the trainees choose to attend is directly related to their PhD dissertation research. The TL1 trainees participate in this clinical experience during the third year of their PhD program since they are well into their dissertation research and usually have no other didactic requirements at that time. At this point, they have also learned how to utilize their time more efficiently. Expected results from participation in the course include the ability to (1) demonstrate a detailed understanding of the pathophysiology and treatment of the disease and (2) demonstrate knowledge of current research literature and provide references (Table 1).

**Table 1.** Guidelines for the Translational Sciences Clinic. The guidelines are provided to the trainee and mentor before the start of the rotation

**Course description**
The Translational Science Clinic is designed for TL1 trainees. Trainees will shadow a clinician–scientist in an outpatient clinic that is directly related to the trainee’s dissertation research. The objectives of this course are to create an environment where trainees begin to develop an appreciation for the process of translational research and to introduce trainees to mentors who can also serve as role models. The course consists of a maximum one-half day per week in a clinic with the same faculty member and will count as two credit hours.
**Student and mentor expectations**
**The trainee should**
Complete the *MyQuest HIPAA training module* prior to signing up for the course
Contact the program director at least one month prior to the clinic to discuss possible clinical mentors. The mentor should have an MD or MD/PhD and work in a clinic related to the trainee’s dissertation topic; the program director or associate program director will provide final approval of the chosen mentor.
Gain familiarity with the clinic on the first clinic visit and then begin to see patients with the mentor.
Dress professionally (no shorts or open-toe shoes) and wear a laboratory coat if she/he has one.
Complete the required evaluation.
**The mentor should**
Assign patients who the trainees will shadow in the clinic.
Introduce the trainees to the staff and expectations of the clinic.
Challenge the trainee’s knowledge of current research literature.
Complete the required evaluation and discuss the responses with the trainee.
**Instructional methodologies and activities**
Trainees will perform literature searches to discover the latest concepts concerning the patients’ disease and discuss them with the clinician–scientist. They are encouraged to write a review article on a topic germane to their research and clinical observations with their mentor, if appropriate.
At the end of the semester, trainees should be able to
Demonstrate a detailed understanding of the pathophysiology and treatment of the disease.
Demonstrate knowledge of current research literature and provide references.
**Grading**
The trainee and mentor will both complete evaluation forms at the end of the semester. The final grade in the course (honors/pass/no pass) is determined by the mentor’s evaluation of the trainee. The mentor must share their evaluation with the trainee, but the trainee is not required to share their evaluation with the mentor.

The Translational Sciences Clinic also serves as a compliment to the TL1 program’s unique journal club [2]. The trainees participate in the Journal Club during their first year in the TL1 program. The Journal Club provides the trainees the opportunity to see the evolution of a basic science discovery through the process to dissemination and implementation. Thus, the Translational Sciences Clinic provides the opportunity to extend what the trainees learned in the Journal Club.

## Methods

Guidelines were established for the rotation (Table 1) and were provided to the trainees and clinical mentors in advance of the trainees coming to the clinic for the first time. The trainees were also required to complete the Health Insurance Portability and Accountability Act (HIPAA) training course before they participated in the clinic. Although they did not have direct access to the patient’s medical history or electronic health record, this course provided essential information about the importance of preserving and implications for breaching patient confidentiality. Since 2016, 27 PhD and dual-degree students have taken the Translational Sciences Clinic as part of the TL1 predoctoral training program. The majority of students (22; 81%) were/are pursuing PhDs from the College of Graduate Studies. Four (15%) were/are part of the dual-degree MD/PhD MSTP from the College of Medicine/College of Graduate Studies and one student (3%) was in the DMD/PhD program from the College of Dental Medicine.

Clinical mentors represented a range of specialties including pediatric hematology/oncology, pediatric infectious diseases, neurology/movement disorders, surgery, oncology/hematology, rheumatology, dental medicine, pulmonology, pediatrics, and psychiatry. The mentors were assistant, associate, or full professors and several were also institutional career development (KL2) scholars. The latter provided some near-peer mentoring. A list of potential clinical mentors was provided to the trainees; however, they could choose a mentor not on the list; mentors were required to have an MD or MD/PhD and work in a clinic related to the trainee’s dissertation topic. In either case, the selection of the clinical mentor had to be approved by either the TL1 program director or associate program director. Other trainee requirements included dressing professionally, acquainting themselves with the clinical operations/guidelines prior to shadowing, and completing the required mentor evaluation. Trainees were also expected to complete literature searches on the disease topics to discuss with the clinical mentor and serve as a review article framework, when appropriate.

Clinical mentors were expected to introduce the trainee to the staff and rules of the clinic, identify patients for the trainee to shadow, confer with the trainee, and furnish a final grade and trainee evaluation (Table 1).

An important component of this training experience was to be able to fully evaluate both the trainees’ and mentors’ experiences. Thus, in 2016, we formulated a series of questions that we felt would provide insight into the perceptions of both the trainee and the mentor. Questions were developed by a multidisciplinary team to assess mentor and mentee perception of progress toward program goals [3] and were evaluated by the team for content, clarity, ease of understanding, usefulness, and comprehensiveness through an iterative process [4]. Between 2018 and 2019, the survey response scale was adapted to assess level of agreement (strongly agree to strongly disagree) rather than assessment of quality (outstanding to poor).

At the end of the semester, the clinical mentors and trainees completed the evaluation forms (Tables 2a and 3a). The surveys were administered anonymously by the program assistant, and responses were compiled for the program director, associate program director, and South Carolina Clinical and Translational Research Institute program coordinator to review (Supplementary Information). The surveys were used for quality improvement/program evaluation purposes and were classified as such by the institutional review board. Therefore, no informed consent was required. The mentor was required to discuss their evaluation with the trainee and provide a final grade. The trainee completed the form but did not have to share their evaluation with the mentor. Both forms were submitted to a central location for review by the program director and associate program director. Evaluations were initially administered using paper forms, and the data were entered into a REDCap database by a TL1 program staff member [5]. Evaluations are now administered solely using REDCap to ensure confidential and secure storage of data.

**Table 2.** Evaluation by the trainees (a) and truncated anecdotal comments from the trainees (b). From 2016 to 2018, trainees provided responses to four evaluation questions using a four-point scale ranging from outstanding (1) to poor (2). In 2019, the same evaluation questions were administered but on a five-point scale (1 – strongly agree, 2 – agree, 3 – undecided, 4 – disagree, and 5 – strongly disagree). Both the trainee and the mentor were encouraged to provide comments at the bottom of the evaluation forms

a. Questions	2016–2018 (*n* = 7)	2019 (*n* = 11)
Q1. My mentor challenged my understanding of the scientific literature.	86% outstanding 14% excellent	82% strongly agree 18% agree
Q2. I learned how to begin to integrate the latest scientific information into concepts of improving understanding and treatment of diseases.	71% outstanding 29% excellent	82% strongly agree 18% agree
Q3. This elective helped me to begin to understand how to integrate basic science into clinical investigation.	100% outstanding	91% strongly agree 9% agree
Q4. This has been a valuable experience.	100% outstanding	91% strongly agree 9% agree
b. Trainees’ comments		
… this experience gave me wonderful insight on how to practice translational medicine and science in order to bridge the gaps between the labs and clinics.		
I have gained an immense appreciation of the ongoing need for readily translatable therapies in preclinical models that can be applied in clinical settings.		
The clinical experience has been invaluable.		
My experience in the clinic has challenged my critical thinking in a meaningful way. It makes me think how to not only make my study understandable at the basic science level but also at the translational level.		
Thank you for the learning opportunity! I also feel that I have a closer connection to the diseases I study by now seeing the patients in person who are fighting these diseases.		
I got a much better idea of what the ailments I am studying actually entail by visiting the clinic.		
This rotation with Dr. N was without doubt the best aspect of the TL1 program, and maybe one of the best experiences in my graduate school training thus far. I don’t think there are enough words to accurately describe how incredible and impactful this rotation has been. This was an incredible rotation!		

**Table 3.** Evaluation by the mentors (a) and mentors’ comments (b). From 2016 to 2018, mentors provided responses to five evaluation questions using a four-point scale ranging from outstanding (1) to poor (2). In 2019, the same evaluation questions were administered but on a five-point scale (1 – strongly agree, 2 – agree, 3 – undecided, 4 – disagree, and 5 – strongly disagree). Both the trainee and the mentor were encouraged to provide comments at the bottom of the evaluation forms

a. Question	2016–2018 (*n* = 9)	2019 (*n* = 9)
Q1. The student was able to effectively integrate current scientific literature into the diagnosis and treatment plan.	75% outstanding 25% excellent	78% strongly agree 22% agree
Q2. The student conducted effective literature searches for the specific disease topic.	56% outstanding 44% excellent	67% strongly agree 33% agree
Q3. The student displayed enthusiasm for integrating current research literature with clinical problems.	100% outstanding	89% strongly agree 11% agree
Q4. The student was able to conceptualize research questions that integrated current literature and disease states.	67% outstanding 33% excellent	Missing – likely perceived as NA
Q5. The student challenged you scientifically in an appropriate manner.	44% outstanding 56% excellent	67% strongly agree 33% agree
b. Mentor’s comments		
• C is very bright, enthusiastic, and organized. We enjoyed having her in clinic.		
• … met regularly with me to discuss the clinical/translational literature on the topic.		
• She would be an excellent addition and asset to any team as she moves forward in her career.		
• D works effectively, efficiently and is very self-motivated. Her presentations are always excellent.		
• S was a great addition to our clinic team.		
• She has a genuine interest in integrating her basic science knowledge with the understanding and treatment of oral disease.		
• X was always well prepared for clinic and came with appropriate and thought provoking questions. It was a pleasure having her in clinic with me and she is welcome anytime.		
• I thought S did a great job.		
• I enjoyed having D in clinic with me. We had interesting discussions.		
• C was enthusiastic about seeing patients and asked great questions. The patients were excited to see him as well.		
• It was a pleasure to have B in the clinic and he integrated very well with the clinic staff and patients. He asked appropriate questions and interacted with the patients at the level of 4th year medical student which is very impressive given his non-clinical training.		
• C did a wonderful job during this rotation.		

## Results

Perhaps, the most rewarding results were the responses of the trainees; they ranked all questions predominantly strongly agree or outstanding (Table 2a). Of particular note was that between 90 and 100% of the trainees responded that this course helped them to better understand incorporating basic science research into clinical care; they also reported a greater appreciation for integrating concepts from scientific literature into disease treatments. As can be seen in the trainees’ anecdotal comments, the Translational Sciences Clinic course was a very valuable experience for them (Table 2b). Interestingly, there were no negative comments about their experiences.

The clinical mentors rated the trainees positively in all categories (Table 3a). Their anecdotal comments reinforced the positive experiences that they had with the trainees (Table 3b). The mentors also had to provide the final grade for the trainees; most received a grade of honors and the rest received a pass. None of the trainees received a no pass grade. We believe that changing the rating scale (2019 cohort) to level of agreement made it easier for the mentors to objectively rate the trainees. The mentors were all asked if they would like to participate in this program again in the future and they said yes.

The mentors represented many medical disciplines and specialties. We purposely did not restrict the mentor pool to any specific discipline since the trainees’ research interests were quite broad.

## Discussion

The positive experiences of both the mentors and trainees motivated our program to create awareness of this unique translational research experience so that it can be adapted at other academic institutions. It should be recognized that the vast majority of trainees were third-year PhD candidates with no prior clinical exposure, yet they were not intimidated by the clinical experience and exposure to patients. Instead, as noted in their comments, they found the Translational Sciences Clinic to be one of the most rewarding experiences of their PhD training. We recognize that the comments that we have selected are anecdotal, but it should be noted that there were no negative comments by the trainees.

The innovation of this clinical rotation experience lies in the fact that PhD trainees had the opportunity to see patients with the diseases upon which they were conducting their research. The unique experience also speaks to National Center for Advancing Translational Science’s (NCATS) mission of catalyzing the generation of innovative methods and technologies that will enhance the development, testing, and implementation of diagnostics and therapeutics across a wide range of human diseases and conditions [1]. As a result of seeing patients with their disease of interest, participating trainees clearly gained a better understanding of how their research could ultimately be translated from bench to bedside.

Another outcome of the Translational Sciences Clinic was the trainees’ exposure to a team-based approach to patient care. Since there are often many professional personnel of diverse backgrounds involved in the care of the patients, the trainees gained a better appreciation of a team-based approach to problem solving (diagnosing and developing a treatment plan) which could be directly applied to their research projects.

The final question on the mentors’ evaluation form was “Would you be willing to mentor a student again?” They all responded affirmatively, further reinforcing the enthusiasm the clinical mentors had for having PhD trainees shadow in their clinics. Many of the students discussed research-related papers with the mentors in the context of the patient’s diagnosis, furthering the concept of translational research from basic science to public health. In summary, the positive outcomes and responses from both students and physician–scientists involved in the course made the Translational Sciences Clinic a highly effective and beneficial course for TL1 trainees. The structures and guidelines for this unique translational research experience could be easily replicated at other academic medical centers for TL1 trainees or PhD students.

## Supplementary material

For supplementary material accompanying this paper visit https://doi.org/10.1017/cts.2020.529. click here to view supplementary material
