# Supplementary material for: The translational sciences clinic: From bench to bedside
Source: J Clin Transl Sci. 2020 Aug 25;5(1):e36. doi: 10.1017/cts.2020.529 (PMC8057442; doi:10.1017/cts.2020.529)
Supplement: Supplementary file 1 [file S2059866120005294sup001.pdf]

Translational Science Clinic Mentor Evaluation

This evaluation determines the student's grade for the course and must be discussed with them.

1) Student Type
   - ◯ MSTP
   - ◯ TL1
   - ◯ Other

2) Mentor First and Last Name __________________________________

3) Student First and Last Name __________________________________

4) Semester
   - ◯ Fall
   - ◯ Spring
   - ◯ Summer

5) Year __________________________________

	Strongly Agree	Agree	Undecided	Disagree	Strongly Disagree
6) The student was able to effectively integrate current scientific literature into the diagnosis and treatment plan.	◯	◯	◯	◯	◯
7) The student conducted effective literature searches for the specific disease topic.	◯	◯	◯	◯	◯
8) The student displayed enthusiasm for integrating current research literature with clinical problems.	◯	◯	◯	◯	◯
9) The student was able to conceptualize research questions that integrated current literature and disease states.	◯	◯	◯	◯	◯
10) The student challenged you scientifically in an appropriate manner.	◯	◯	◯	◯	◯

11) Overall Evaluation
   - ◯ Honors
   - ◯ Pass
   - ◯ Fail

12) Comments __________________________________

Translational Science Clinic Student Evaluation

1) Student Type

◯ MSTP
◯ TL1
◯ Other

2) Student First and Last Name

____

3) Mentor First and Last Name

____

4) Semester

◯ Fall
◯ Spring
◯ Summer

5) Year

____

	Strongly Agree	Agree	Undecided	Disagree	Strongly Disagree
6) My mentor challenged my understanding of the scientific literature.	◯	◯	◯	◯	◯
7) I learned how to begin to integrate the latest scientific information into concepts of improving understanding and treatment of diseases.	◯	◯	◯	◯	◯
8) This elective helped me to begin to understand how to integrate basic science into clinical investigation.	◯	◯	◯	◯	◯
9) This has been a valuable experience.	◯	◯	◯	◯	◯

10) Comments

____

13) I would be interested in mentoring another student in the future
◯ Yes
◯ No

14) I have discussed this evaluation with the student
◯ Yes
◯ No
